# Incorporating predicted functions of nonsynonymous variants into gene-based analysis of exome sequencing data: a comparative study

**DOI:** 10.1186/1753-6561-5-S9-S20

**Published:** 2011-11-29

**Authors:** Peng Wei, Xiaoming Liu, Yun-Xin Fu

**Affiliations:** 1 Division of Biostatistics, University of Texas School of Public Health, 1200 Herman Presser Drive, Houston, TX 77030, USA; 2 Human Genetics Center, University of Texas School of Public Health, 1200 Herman Presser Drive, Houston, TX 77030, USA

## Abstract

Next-generation sequencing has opened up new avenues for the genetic study of complex traits. However, because of the small number of observations for any given rare allele and high sequencing error, it is a challenge to identify functional rare variants associated with the phenotype of interest. Recent research shows that grouping variants by gene and incorporating computationally predicted functions of variants may provide higher statistical power. On the other hand, many algorithms are available for predicting the damaging effects of nonsynonymous variants. Here, we use the simulated mini-exome data of Genetic Analysis Workshop 17 to study and compare the effects of incorporating the functional predictions of single-nucleotide polymorphisms using two popular algorithms, SIFT and PolyPhen-2, into a gene-based association test. We also propose a simple mixture model that can effectively combine test results based on different functional prediction algorithms.

## Background

Despite the great success of genome-wide association studies (GWAS) in identifying hundreds of loci harboring common single-nucleotide polymorphisms (SNPs) that are associated with complex diseases, most common SNPs identified to date have small effect sizes and the proportion of heritability explained is at best modest for most traits. Thus investigators have become interested in low-frequency or rare variants (minor allele frequency [MAF] < 1%) that may contribute to genetic risk [1]. Recent advances in next-generation sequencing technologies have made it possible, at a relatively low cost, to extend association studies to low-frequency and rare variants, particularly in targeted resequencing of candidate genes or the whole exome.

The statistical power to detect disease association with an individual rare variant is limited, partly because of the small number of observations for any given variant and partly because of the high frequency of sequencing errors. In response to this challenge, several new and powerful statistical methods have been proposed recently, including the combined multivariate and collapsing (CMC) method of Li and Leal [2], the weighted-sum method of Madsen and Browning [3], and the variable threshold (VT) approach of Price et al. [4]. Despite different statistical models, a common strategy adopted by these methods is to group the variants according to function, such as genes and pathways, and compare the group counts or distributions rather than the counts for each variant in the group. The rationale behind this grouping strategy is that if many different mutations in a group affect disease risk, then it may be beneficial to focus on the group rather than on each variant individually.

The VT method of Price et al. [4] is of particular interest because, in contrast to a prespecified threshold for defining rare variants in the CMC method, it allows the allele frequency threshold to vary and thus adapts to properties of individual genes. It is motivated by the fact that some genes may harbor functional alleles at higher frequencies, whereas other genes may have only private functional variants. Another feature of the VT method is that it can incorporate computational predictions of the functional effects of nonsynonymous variants (e.g., by PolyPhen-2 [5]) into the association test, thereby avoiding the loss of power that results from combining both functional and nonfunctional alleles, as in previous grouping methods. The VT method is more powerful than the CMC and the weighted-sum methods for analyzing simulated and empirical sequencing data.

We note that Price et al. [4] used and studied only functional predictions from PolyPhen-2. However, several other algorithms are available for computationally predicting functions of nonsynonymous variants, such as the “sorting tolerant from intolerant” (SIFT) algorithm of Kumar et al. [6], MutationTaster of Schwarz et al. [7], and the “screening for nonacceptable polymorphisms” (SNAP) algorithm of Bromberg et al. [8]. It is yet unclear how the results of different functional prediction-algorithm-based VT tests compare with each other. The objective here is to use the Genetic Analysis Workshop 17 (GAW17) simulated mini-exome data to compare the results of the VT test incorporating predicted functions of nonsynonymous variants from two popular algorithms, PolyPhen-2 and SIFT. Although previous investigators have compared the accuracy of the two algorithms in predicting deleterious mutations (e.g., Flanagan et al. [9] and Adzhubei et al. [5]), we are the first, to our knowledge, to study the effects of incorporating functional predictions based on different computational algorithms in the context of association tests of sequencing data. In addition, we propose a simple mixture model to combine the test results based on different functional prediction algorithms.

## Methods

### Data description

We analyze the simulated mini-exome data set provided by GAW17. This data set consists of a collection of 697 unrelated individuals and their genotypes and phenotypes. The subjects are from the 1000 Genomes Project (http://www.1000genomes.org). There are 24,487 SNPs, among which 13,572 are nonsynonymous, mapped to the exons of 3,205 genes. Two hundred replicates of the phenotype simulation were carried out based on some simulating model, and three quantitative traits and a qualitative trait were available. See Blangero et al. [10] for simulation details. In this study, we analyze only the qualitative trait, that is, disease status, from replicate 1. There were 209 case subjects and 488 control subjects. Because we focus on a gene-based association test, we restrict our analysis only to genes with at least two SNPs, resulting in 1,979 genes and 23,261 SNPs, among which 13,086 are nonsynonymous. The summary statistics of the number of SNPs that each of the 1,979 genes has are as follows: minimum = 2, 25th percentile = 3, median = 6, 75th percentile = 15, and maximum = 231.

### SIFT and PolyPhen-2 algorithms

The SIFT algorithm is a multistep, sequence-homology-based algorithm that classifies amino acid substitutions resulting from nonsynonymous SNPs. The underlying premise for the SIFT algorithm is based on the evolutionary conservation of the amino acids within protein families: Highly conserved positions tend to be intolerant to substitutions, whereas those with a low degree of conservation tolerate most substitutions [6]. The SIFT algorithm predicts that a nonsynonymous variant will be damaging if the scaled probability score, also termed the SIFT score, is less than 0.05; otherwise, the algorithm predicts that the variant will be tolerated.

In contrast to the SIFT algorithm, which does not use the protein structure information, the PolyPhen-2 algorithm uses a naïve Bayes classifier to predict damaging effects of nonsynonymous variants based on eight sequence-based and three structure-based predictive features [5]. The PolyPhen-2 algorithm calculates the naïve Bayes posterior probability that a given mutation will be damaging and qualitatively predicts that it will be benign, possibly damaging, or probably damaging, corresponding to posterior probability intervals [0, 0.2], (0.2, 0.85), and [0.85, 1], respectively.

We obtained the predicted functional scores of all 13,572 nonsynonymous SNPs by means of the online versions of the SIFT algorithm (http://sift.jcvi.org/index.html) and the PolyPhen-2 algorithm (http://genetics.bwh.harvard.edu/pph2/). For both algorithms, we used human genome build 36 from the National Center for Biotechnology Information (NCBI) as the reference genome sequence. For the PolyPhen-2 algorithm, HumDiv was selected as the classifier model because it was recommended for evaluating rare alleles at loci potentially involved in complex phenotypes [5].

### Variable threshold test

In the VT test, rare alleles are grouped together by optimizing an allele frequency threshold that maximizes the difference, as quantified by a *z*-score, between distributions of trait values or disease status for individuals with and without rare alleles. To control type I error, we applied the same optimization procedure to permuted data to obtain an exact *p*-value for association. The rationale underlying the VT method is that for each gene there is some unknown threshold *T* for which variants with a MAF less than *T* are substantially more likely to be functional than those with a MAF greater than *T.* Specifically, for a given gene with *m* SNPs in its exons, we define the *z*-score for a given threshold *T* as:(1)

Where  is an indicator variable that is equal to 1 if the MAF of SNP *i* is less than the threshold *T* and equal to 0 otherwise, *C_ij_* is the reference allele count of SNP *i* in subject *j*, *π_j_* is the phenotype of subject *j* equal to 0 and 1 for control subjects and case subjects, respectively,  is the mean value of *π_j_* across subjects *j*, and *S_i_* is the functional prediction score of SNP *i*, which is between 0 and 1 (larger values indicate higher probability of damaging effect). In addition, the maximum *z*-score is defined as:(2)

The statistical significance of *z*_max_ is then assessed by permutations on phenotypes. In addition, the VT test has been implemented as an R function, available at http://genetics.bwh.harvard.edu/rare_variants/.

### Incorporating the predicted functions of variants into the VT test

To study and compare the effects of incorporating different predicted functions of SNPs into a gene-based association test, we carried out four versions of the VT test: (1) an unweighted VT test, in which all SNPs, both synonymous and nonsynonymous, were grouped (thus *S_i_* in Eq. (1) was 1 for all SNPs); (2) a binary weight VT test, in which only nonsynonymous SNPs were grouped (thus *S_i_* was 1 for nonsynonymous SNPs and 0 otherwise); (3) a SIFT-based VT test, in which *S_i_* was equal to (1 − SIFT prediction score) for nonsynonymous SNPs and 0 otherwise; and (4) a PolyPhen-2-based VT test, in which *S_i_* was equal to the PolyPhen-2 score for nonsynonymous SNPs and 0 otherwise. For those nonsynonymous SNPs without a prediction score, we imputed them with the corresponding median scores: 0.1 for the SIFT algorithm and 0.2 for the PolyPhen-2 algorithm. For each gene, 10,000 permutations were carried out to obtain the *p*-value.

### Mixture model for combining test results

Here, we propose a simple mixture model to combine *p*-values resulted from association tests based on different functional prediction algorithms. Let *p_g_*_1_ and *p_g_*_2_ be gene *g*’s VT test *p*-values corresponding to the SIFT and PolyPhen-2 algorithms, respectively, for *g* = 1, …, *G*. Define the *z*-transformation:(3)

so that smaller *p*-values correspond to larger *z*-values, where Φ^−1^ is the inverse cumulative distribution function of *N*(1, 0) and *k* = 1, 2. We assume that (*x_g_*_1_, *x_g_*_2_) follows a two-component bivariate normal mixture model, that is, that its density is given by:(4)

where *f*_0_ and *f*_1_ are two bivariate normal densities corresponding to *z*-values of non-phenotype-associated and phenotype-associated genes, respectively. The two-component normal mixture model is a simple yet powerful statistical method for genome-wide discoveries [11]. The posterior probability of gene *g* being associated with the phenotype is given by:(5)

which can be used to rank genes and to estimate the false discovery rate (FDR) for a given cutoff for claiming significant genes and thus to control the FDR at a desired level, for example, 5% [12]. For simplicity, we further assume that *x_g_*_1_ and *x_g_*_2_ are conditionally independent given whether gene *g* is associated with the phenotype or not; that is,(6)

where *ϕ*(*x*; *μ*, *σ*^2^) is the density function of *N*(*μ*, *σ*^2^) and *l* = 0, 1. The conditional independence mixture model is similar to a naïve Bayes method except that the mixture model is unsupervised learning, whereas the Bayes method is supervised learning. Note that this simplified model may not provide goodness-of-fit to the *z*-values, and thus the resulting posterior probabilities can only be used to rank genes, not to estimate the FDR (see Wei and Pan [13]). The parameter estimates in the normal mixture model can be obtained by means of the EM algorithm, which is implemented in the R package mclust. In addition, *p*-values from a single type of association test, for example, the SIFT-based VT test, can be used to fit a two-component univariate normal mixture model and the FDR can be similarly estimated.

## Results

### Prediction score comparison: SIFT vs. PolyPhen-2 algorithms

**Figure 1:**
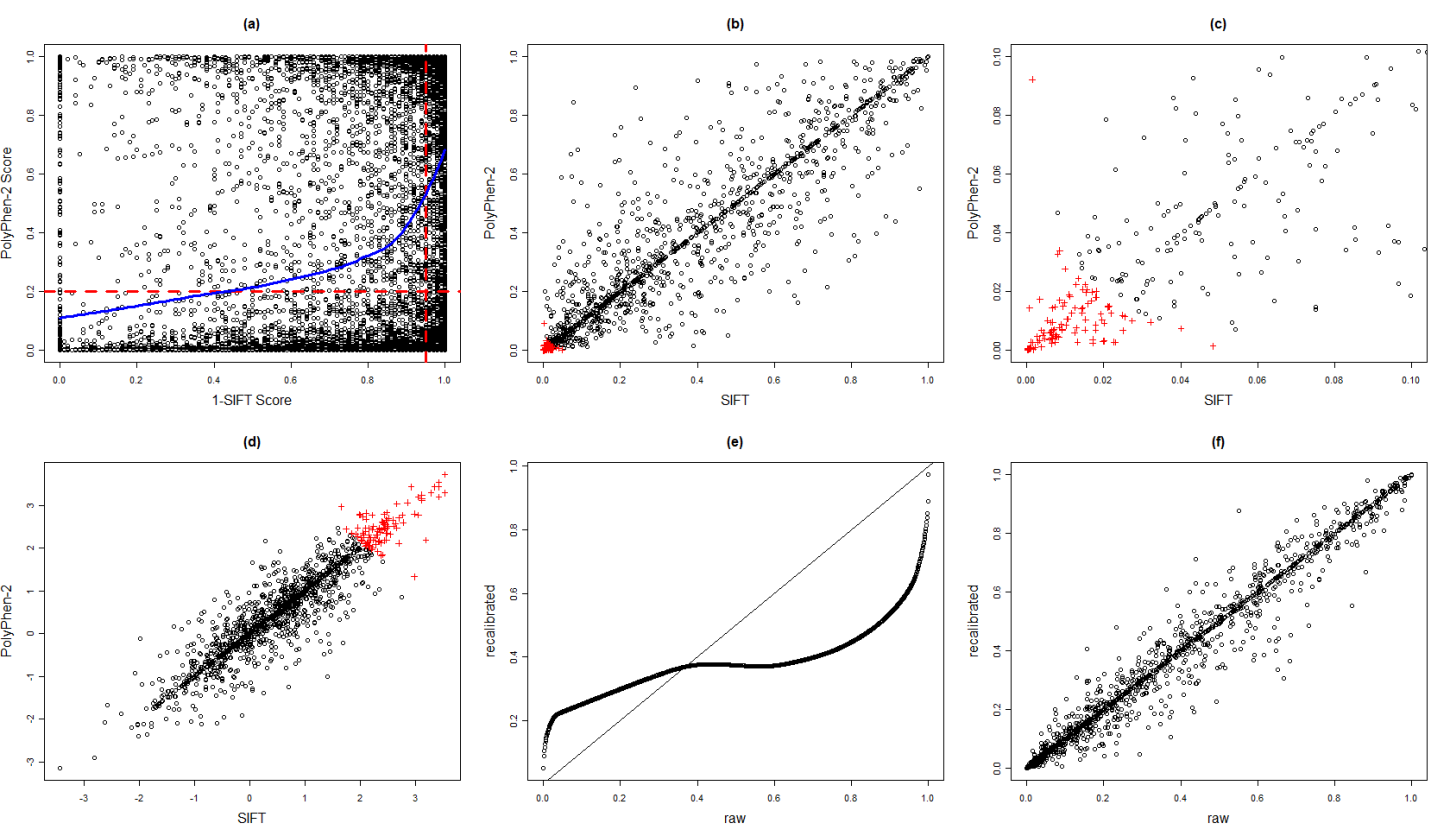
**SIFT scores versus PolyPhen-2 scores.** (a) (1 − SIFT score) plotted against PolyPhen-2 score. The red dashed lines correspond to the thresholds for predicting deleterious variants: 0.95 for SIFT and 0.2 for PolyPhen-2. The blue solid line corresponds to the LOESS curve (locally weighted scatterplot smoothing). (b) SIFT-based VT test *p*-values plotted against PolyPhen-2-based VT test *p*-values. Red plus signs correspond to genes that had tied rank 1 (posterior probabilities of association equal to 1) by the mixture model combining both tests. (c) Enlarged section of part b. (d) SIFT-based VT test *z*-values plotted against PolyPhen-2-based VT test *z*-values. Red plus signs correspond to genes that had tied rank 1 by the mixture model combining both tests. (e) Raw versus recalibrated PolyPhen-2 scores; solid line is the identical line. (f) Raw versus recalibrated PolyPhen-2 score-based VT test *p*-values.

As described in the Methods section, we obtained the prediction scores of being deleterious using the SIFT and PolyPhen-2 algorithms for the 13,572 SNPs annotated as nonsynonymous in the annotation file supplied by GAW17. Nine hundred thirty-nine nonsynonymous SNPs did not have a SIFT score and 1,241 nonsynonymous SNPs did not have a PolyPhen-2 score, probably because of gene annotation errors or insufficient sequence evidence. Note that nonsynonymous variants with a PolyPhen-2 score larger than 0.2 were predicted to be possibly or probably damaging, whereas those with a SIFT score less than 0.05 were predicted to be damaging. As a result, we plotted the (1 − SIFT score) against the PolyPhen-2 score in Figure 1a. The scatterplot together with the LOESS curve shows that the two scores are positively correlated, although there are quite a few SNPs with discordant prediction scores. We also assessed the correlation of dichotomous predictions from the two algorithms. Using 0.2 and 0.95 as thresholds for the PolyPhen-2 and SIFT scores, respectively, we obtained a two-by-two table with cell counts as follows: P+ and S+ = 3,600, P− and S− = 4,492, P+ and S − = 2,403, and P− and S+ = 1,345. This resulted in an odds ratio (OR) estimate equal to 5 (chi-square test *p* < 10^−16^), meaning that the odds of being predicted to be deleterious using the PolyPhen-2 algorithm for variants that were predicted to be deleterious using the SIFT algorithm were five times the odds for those that were predicted to be benign using the SIFT algorithm. Similar comparison results held for the 13,086 nonsynonymous SNPs corresponding to the 1,979 genes with at least two SNPs.

### Comparison of SIFT-based and PolyPhen-2-based VT tests

Figures 1b-d compare the *p*-values and *z*-values of SIFT-based and PolyPhen-2-based VT tests. Although the two *p*-values are positively correlated overall, they can be substantially different from each other. However, smaller *p*-values seem to be better correlated, as demonstrated by the upper-right part of the *z*-value plot Figure 1d. In addition, we fitted a two-component bivariate normal mixture model to combine the *p*-values of the two tests, as described in the Methods section. One hundred sixty genes were ranked 1 (i.e., the posterior probabilities of association were all equal to 1) in the combined analysis and were plotted as red plus signs in Figures 1b-d. Not only were genes with small *p*-values highly ranked, but genes with moderately small *p*-values could also be boosted to have a tied rank of 1 (Figure 1c).

**Figure 2:**
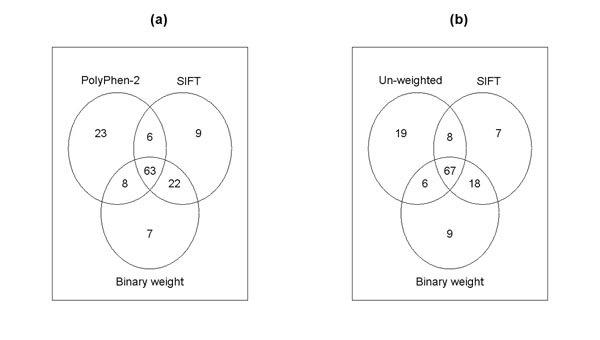
**Venn diagrams for the top 100 genes.** Top 100 genes found by (a) PolyPhen-2, SIFT, and binary-weight-based VT tests and (b) unweighted, SIFT, and binary-weight-based VT tests.

In addition to the comparison between SIFT-based and PolyPhen-2-based tests, we also performed comparisons among all four versions of the VT test. Specifically, we looked at the overlaps among the top 100 genes by each test, as shown by the Venn diagrams in Figure 2. We can see that the SIFT-based and the binary weight-based tests share a large number of genes, whereas the PolyPhen-2-based and the unweighted tests share much fewer genes with the former two tests. This comparison also suggests, however, that association tests incorporating different functional predictions could lead to quite different results. In practice, it is unlikely that one functional prediction algorithm will be dominantly better than the other, which necessitates a combined analysis in an effective way, such as the mixture model proposed here. In addition, Table 1 lists the top 10 genes by the SIFT-based VT test, all of which were tied at rank 1 by the combined analysis. All genes had small *p*-values, as ascertained by the other three tests, as well as a large number of SNPs sufficiently representing the corresponding genes.

**Table 1:** Top ten genes ranked by SIFT-based VT test *p*-value

Gene	SIFT	PolyPhen-2	Binary	Unweighted	Number of SNPs	Number of nonsynonymous SNPs
*FAM13A1*	0.0002	0.0001	0.0007	0.0003	34	23
*DGKZ*	0.0002	0.0005	0.0002	0.0004	22	15
*TRIM42*	0.0003	0.0002	0.0009	0.0032	39	30
*ADAM15*	0.0003	0.0003	0.0016	0.0004	30	20
*FLT1*	0.0003	0.0007	0.0002	0.0002	35	20
*GRIA4*	0.0004	0.0003	0.0004	0.0066	18	6
*IRF6*	0.0005	0.0005	0.0021	0.0119	15	7
*HDAC4*	0.0007	0.0144	0.0011	0.0010	36	16
*GDF15*	0.0009	0.0006	0.0040	0.0006	10	6
*SUSD2*	0.0009	0.0008	0.0015	0.0005	45	29

All genes had tied rank 1 by the mixture model combining both SIFT-based and PolyPhen-2-based VT test *p*-values. *P*-values were obtained from 10,000 permutations.

### Comparison of raw and recalibrated PolyPhen-2 scores

Price et al. [4] suggested that, to obtain optimal results, the PolyPhen-2 scores should be recalibrated before being applied to the VT test. We obtained the recalibrated PolyPhen-2 scores using the computer program provided by Price et al. [4]. Figure 1e shows the raw versus the recalibrated PolyPhen-2 scores, which were calculated using a nonlinear monotone transformation of the raw scores. In addition, the VT test *p*-values based on the raw and recalibrated PolyPhen-2 scores are compared in Figure 1f. Although the *p*-values are highly correlated with Spearman’s rank correlation coefficient equal to 0.98, they could be quite different for some genes.

## Discussion

In the present analyses, we compared the raw and recalibrated PolyPhen-2 scores in the VT test. It would also be of interest to develop methods for recalibrating the SIFT scores; however, this would necessitate having available credible neutral and damaging nonsynonymous SNPs as a training set to derive the recalibration transformation. Another possible direction for future investigation is to develop association tests that are more robust to misspecifications of functional predictions and can incorporate covariate effects including environmental factors.

## Conclusions

Motivated by the fact that many algorithms for predicting damaging effects of nonsynonymous variants are available, we performed a comparative study of the effects of incorporating different functional predictions into association tests using the GAW17 simulated mini-exome data set. Our study reveals that, although the PolyPhen-2 and SIFT prediction scores are positively correlated overall, they can be substantially different from each other, quantitatively as well as qualitatively. As a result, the SIFT-based and the PolyPhen-2-based VT test results can also differ. Importantly, our analyses suggest that the two-component normal mixture model proposed here provides a probabilistic approach to effectively combining the heterogeneous test results. Further refinements, including relaxing the conditional independence assumption to improve the goodness-of-fit, are needed.

## Competing interests

The authors declare that there are no competing interests.

## Authors’ contributions

PW conceived and designed the study, performed the statistical analyses and drafted the manuscript. XL co-designed the study. All authors helped to draft the manuscript. All authors read and approved the final manuscript.
